# Electrochemical determination of fenitrothion pesticide based on ultrathin manganese oxide nanowires/molybdenum titanium carbide MXene ionic nanocomposite and molecularly imprinting polymer

**DOI:** 10.1007/s00604-024-06320-5

**Published:** 2024-04-02

**Authors:** Bahar Bankoğlu Yola, Gül Kotan, Onur Akyıldırım, Necip Atar, Mehmet Lütfi Yola

**Affiliations:** 1 Department of Engineering Basic Sciences, Faculty of Engineering and Natural Sciences, Gaziantep Islam Science and Technology University, Gaziantep, Turkey; 2 Department of Chemistry and Chemical Processing Technologies, Kars Vocational School, Kafkas University, Kars, Turkey; 3 Department of Chemical Engineering, Faculty of Engineering and Architecture, Kafkas University, Kars, Turkey; 4 Department of Chemical Engineering, Faculty of Engineering, Pamukkale University, Denizli, Turkey; 5 Department of Nutrition and Dietetics, Faculty of Health Sciences, Hasan Kalyoncu University, Gaziantep, Turkey

**Keywords:** Fenitrothion, Sensor, Voltammetry, Imprinting, Electropolymerization

## Abstract

A novel molecularly imprinted electrochemical sensor is presented based on one-dimensional ultrathin manganese oxide nanowires/two-dimensional molybdenum titanium carbide MXene (MnO_2_NWs@Mo_2_TiC_2_ MXene) for fenitrothion (FEN) determination. After the synthesis of MnO_2_NWs@Mo_2_TiC_2_ MXene ionic nanocomposite was successfully completed with a facile hydrothermal and the pillaring methods, a new type molecular imprinted electrochemical sensor based on MnO_2_NWs@Mo_2_TiC_2_ MXene was constructed with cyclic voltammetry (CV) polymerization including pyrrole monomer and FEN target molecule. After the characterization studies including spectroscopic, electrochemical and microscopic methods, the analytical applications of the prepared sensor were performed. A linearity of 1.0×10^−9^–2.0×10^−8^ mol L^−1^ was obtained and the values of the quantification limit (LOQ) and the detection limit (LOD) were 1.0×10^−9^ mol L^−1^ and 3.0×10^−10^ mol L^−1^, respectively. The studies of selectivity, stability and reproducibility of the constructed sensor based on MnO_2_NWs@Mo_2_TiC_2_ nanocomposite and molecularly imprinting polymer (MIP) were carried out in detail. Finally, the developed sensor was applied to white flour samples with the values close to 100%.

**Graphical Abstract:**

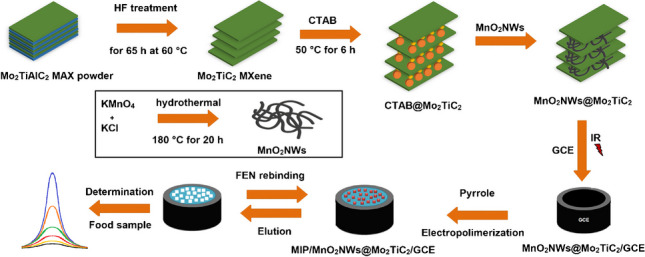

**Supplementary Information:**

The online version contains supplementary material available at 10.1007/s00604-024-06320-5.

## Introduction

After the mid-twentieth century, a significant reduction in agricultural areas has occurred worldwide. Consequently, the applications of synthetic fertilizers and agricultural pesticides have gained momentum to improve yield per unit area in diminishing agricultural land [1]. As an agricultural medicine, the pesticides are the used chemicals to protect agricultural products from harmful organisms and increase productivity. However, the contamination of these chemicals in foods and their adverse effects on consumer health are becoming a matter of daily concern [2]. The acute effects of pesticides on human health can range from skin problems or irritation of the respiratory tract, such as the nose and throat, to systemic effects that can lead to fatality. Pesticides also contribute to the contamination of surface waters. Irrigations on agricultural lands or rainfall can lead to the transfer of these chemicals into surface waters. Consequently, the organisms living in contaminated water sources are frequently exposed to pesticides [3–5]. FEN, classified as a moderately toxic pesticide, is an organophosphorus insecticide and it has been used since 1959 for the insect control in fruits and vegetables. According to World Health Organization, the acceptable limit of FEN has been 0.3–0.5 mg kg^−1^ in fruits and vegetables and it is banned in most European countries [6]. Especially, the usage of flour in the bread preparation poses a significant risk due to the potential presence of FEN pesticide residues. Processing steps such as grinding and cooking have been noted to substantially reduce FEN residues in wheat flour. Some studies indicate that bread made from white flour contains higher levels of FEN residues [7, 8]. Hence, the presence of pesticide residues in the flour causes the contamination of animal products and, consequently, impacting individuals at the top of the food chain. Therefore, the identification of potential pesticide residues in white flour is crucial for assessing risks that may affect a diverse range of food items.

MXene having 2D nanosheets is formulated as Mn^+1^X_n_T_x_ (M: transition metal, X: carbon/nitrogen, Tx: surface termination group including hydroxyl, fluorine and oxygen and *n* = 1, 2 or 3). Moreover, MXenes can be grouped into different classes such as single transition metal or multiple transition metal based MXenes [9, 10]. MXenes have superior physical properties including high conductivity, high stability and hydrophilicity, and thus their superior hydrophilic features and low diffusion barriers can provide the potential electrode materials for electrochemical sensor applications [11–13]. Nonetheless, MXenes’ restacking property in sensor applications can limit MXenes’ efficient usage in practical applications. Several methods have been utilized to eliminate the nanosheets’ restacking and to increase the electrochemical sensor performance. The construction of the curved 2D nanosheets is a known method to solve this restacking problem. In this method, the access to parallel channels may not be sufficient [14]. The other approach is the nanosheets’ vertical growth on substrate via chemical vapor deposition, providing the parallel channels. Nonetheless, the deposition of 2D nanosheets may not be sufficient and the capacitive performance can be reduced [15]. In recent years, the important approach to increase 2D MXenes’ performance is the interlayer spacers incorporation into the nanosheets [16]. The intercalation of MXenes with transition metal oxide can increase the electrochemical performance and capacitance in the preparation of electrode materials [17]. The different metal oxides such as LaFeO_3_ and ZnO can be used for the intercalation agent [18, 19]. In addition, manganese dioxide (MnO_2_) is one of the most used transition metal oxide materials due to its capacitive performance, high theoretical capacity and environment-friendly nature [20].

Molecular imprinting is a simple technique for the production of template molecule-specific polymeric materials. This technique suggests the certain nano-cavities in compatibility in terms of structure, size and chemical functions [21]. According to this process, the intermolecular interactions occur between functional groups, such as between enzyme-substrate or antibody-antigen. The suitable monomers, cross-linkers, template and the species containing the target molecule are present together in the molecular imprinting method [22, 23]. In general, the molecular imprinting method basically consists of three steps: (i) pre-complexation, (ii) polymerization such as UV and electro-polymerization and (iii) removal of the template (target) molecule from the polymeric network [21, 24]. In the literature, we see that MIP-based techniques were used for FEN separation and recognition. For instance, the molecularly imprinted solid-phase extraction method, using ethacrylic acid as functional monomer and ethyleneglycol dimethacrylate as crosslinking agent, was developed and 59% extraction efficiency was achieved [25]. In other study, a screen-printed sensor based on MIP was presented for FEN detection in forest samples. The polymeric film was developed by CV electro-polymerization of Ni(II)-phtalocyanine and the linear range of 3.0×10^−6^ to 3.0×10^−4^ mol L^−1^ with a LOD of 8.0×10^−7^ mol L^−1^ was obtained with high selectivity [26]. Finally, MIP based on multi-walled carbon nanotubes was synthesized for micro-solid-phase extraction of FEN and the developed method showed a linearity of 5.0–220.0 μg L^−1^ with a detection limit of 1.30 μg L^−1^ [27]. Thus, molecular imprinting technology appears to be frequently used and important for FEN analysis and recognition.

In this research, a new type molecularly imprinted electrochemical sensor based on one-dimensional ultrathin manganese oxide nanowires/two-dimensional molybdenum titanium carbide MXene nanocomposite was presented for an organophosphorus pesticide fenitrothion determination. Because of the long-tail CTA^+^ cationic surfactant’s easy intercalation with MnO_2_NWs between the layers of MXene, CTAB was utilized for the pre-pillaring of MXene. In addition, it could form a homogeneous MXene solution in contrast to ultra-pure water. MnO_2_NWs’ low conductivity could be improved via the formation of 1D/2D heterostructure including MXene. Thus, the pesticide detection will be made faster and more accurately by the developed MIP-based sensor, and the significant concerns about safe food consumption will disappear in the future.

## Experimental

### Chemicals

FEN, methyl parathion (METP), malathion (MAL), vinclozolin (VIN), hydroquinone (HQ), Mo_2_TiAlC_2_ MAX, hydrofluoric acid (HF), potassium permanganate (KMnO_4_), potassium chloride (KCl), cetyltrimethylammonium bromide (CTAB) and pyrrole (Py) monomer were purchased from Sigma-Aldrich Merck Group company (St. Louis, Missouri, USA). Phosphate-buffered saline (pH 4.5, 0.1 mol L^−1^ PBS) was selected as a supporting electrolyte.

### Instrumentation

The used instruments for analytical and structural analyzes were given in Supplementary Data. GAMRY Reference 600 workstation was also used for electrochemical works such as electrochemical impedance spectroscopy (EIS), square wave voltammetry (SWV) and cyclic voltammetry (CV). Small amounts of the prepared samples and the whole instruments for characterization studies were stored in closed environments to avoid being affected by temperature and pressure changes.

### Synthesis of Mo_2_TiC_2_MXene*, *MnO_2_NWsand MnO_2_NWs@Mo_2_TiC_2_ nanocomposite

Mo_2_TiAlC_2_ MAX powder (2.0 g) was firstly suspended in 50 wt% concentrated HF solution (15.0 mL) for 65 h at 60°C in an oil bath. Then, Mo_2_TiC_2_ MXene was obtained by the centrifugation and washed with ultra-pure water three times at 4000 rpm. Lastly, Mo_2_TiC_2_ MXene was collected by filtration and dried at 80°C for 12 h.

A facile hydrothermal method was used for the preparation of MnO_2_NWs with high purity and large crystallinity [28]. KMnO_4_ (240.0 mg) and KCl (40.0 mg) were added in ultra-pure water (1.0 L) and stirred for 40 min at 25°C. After that, the suspension was transferred to an autoclave at 180°C for 20 h. After 20 h, the resultant product was washed with ultra-pure water three times and MnO_2_NWs was collected by filtration and dried at 80°C for 12 h.

A facile liquid-phase pre-pillaring and pillaring techniques were utilized for the preparation of MnO_2_NWs@Mo_2_TiC_2_ nanocomposite to provide the high permanent porosity, thermal stability and catalytic feature [29]. Mo_2_TiC_2_ MXene (50.0 mg) was suspended in 0.2 wt% CTAB solution (50.0 mL) 50°C for 6 h. After that, MnO_2_NWs (50.0 mg) was sonicated in ultra-pure water (50.0 mL) and transferred slowly in the prepillared CTAB@Mo_2_TiC_2_ suspension for 15 min. Thus, the resulting product MnO_2_NWs@Mo_2_TiC_2_ nanocomposite was washed with ultra-pure water three times and dried at 80°C for 12 h [30].

### Preparation of MnO_2_NWs@Mo_2_TiC_2_ modified glassy carbon electrode (MnO_2_NWs@Mo_2_TiC_2_/GCE)

The cleaning procedure of the glassy carbon electrode (GCE) surface was given in detail in our previous work [31]. After the cleaning procedure, aqueous MnO_2_NWs@Mo_2_TiC_2_ suspension (30.0 μL, 0.30 mg mL^−1^) was dropped on the clean GCE and dried under IR lamp (MnO_2_NWs@Mo_2_TiC_2_/GCE). The other two modified electrodes such as Mo_2_TiC_2_ MXene/GCE and MnO_2_NWs/GCE were prepared by using the same modification procedure.

### Development of FEN imprinted electrode and the removal from electrode surface

**Scheme 1:**
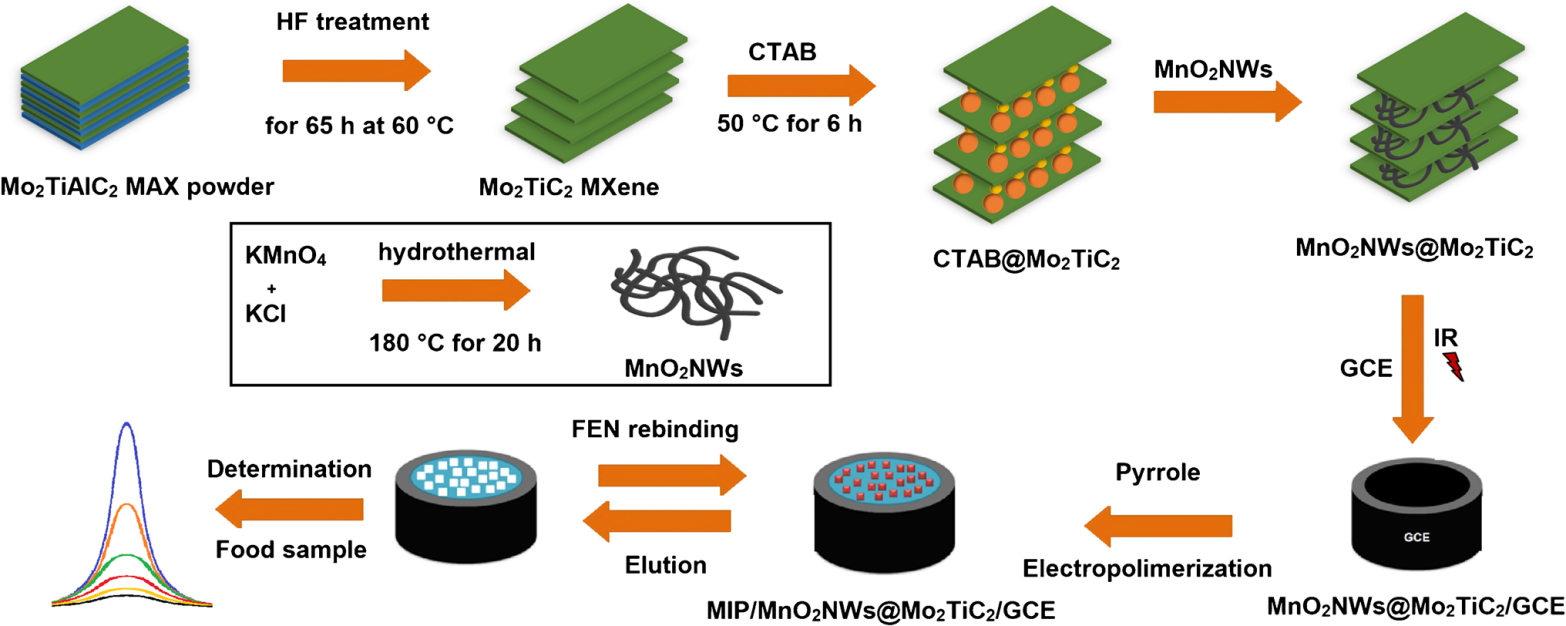
Protocol of MnO_2_NWs@Mo_2_TiC_2_/GCE nanocomposite preparation and MIP/MnO_2_NWs@Mo_2_TiC_2_/GCE development

After the 25.0 mmol L^−1^ FEN and 100.0 mmol L^−1^ Py monomer solution prepared in 0.1 mol L^−1^ PBS was transferred to the electrochemical cell, the nitrogen gas was passed to remove dissolved oxygen for 10 min. After 10 min, the polymerization peaks occurring at approximately +0.70 V were monitored by applying a high potential in the 0.0/+1.0 V potential range by CV method. CV method was used to create FEN-imprinted nano-cavities on the electrode surface. After 25 scans, the electrode was removed from the electrochemical cell. After the prepared electrode was washed with ultra-pure water three times and stored at 25°C (MIP/MnO_2_NWs@Mo_2_TiC_2_/GCE). The preparation protocol of NIP/MnO_2_NWs@Mo_2_TiC_2_/GCE was applied by using the same procedure above without FEN molecule. Scheme 1 demonstrated the protocol of MnO_2_NWs@Mo_2_TiC_2_/GCE nanocomposite preparation and MIP/MnO_2_NWs@Mo_2_TiC_2_/GCE development.

0.1 mol L^−1^ NaCl was used as the desorption solution to break the electrostatic/hydrogen bond interactions between the FEN molecule and the monomer on the electrode surface. For this removal procedure, the electrode was placed in a conical flask containing 0.1 mol L^−1^ NaCl (25.0 mL). Then, the electrode was kept in the shaking bath system for 20 min and the electrode was dried at 25°C.

### Sample preparation

White flour samples (1.0 g), purchased from a local market, were suspended in a mixture of ethyl alcohol:ultra-pure water (20.0 mL, 1:1, v/v) and then centrifuged (5 min at 10000 rpm). After the centrifugation process, the clear part was transferred to another tube and diluted with 0.1 mol L^−1^ PBS (pH 4.5) to fall within the calibration range.

## Results and discussion

### Characterizations of the synthesized nanomaterials

**Fig. 1:**
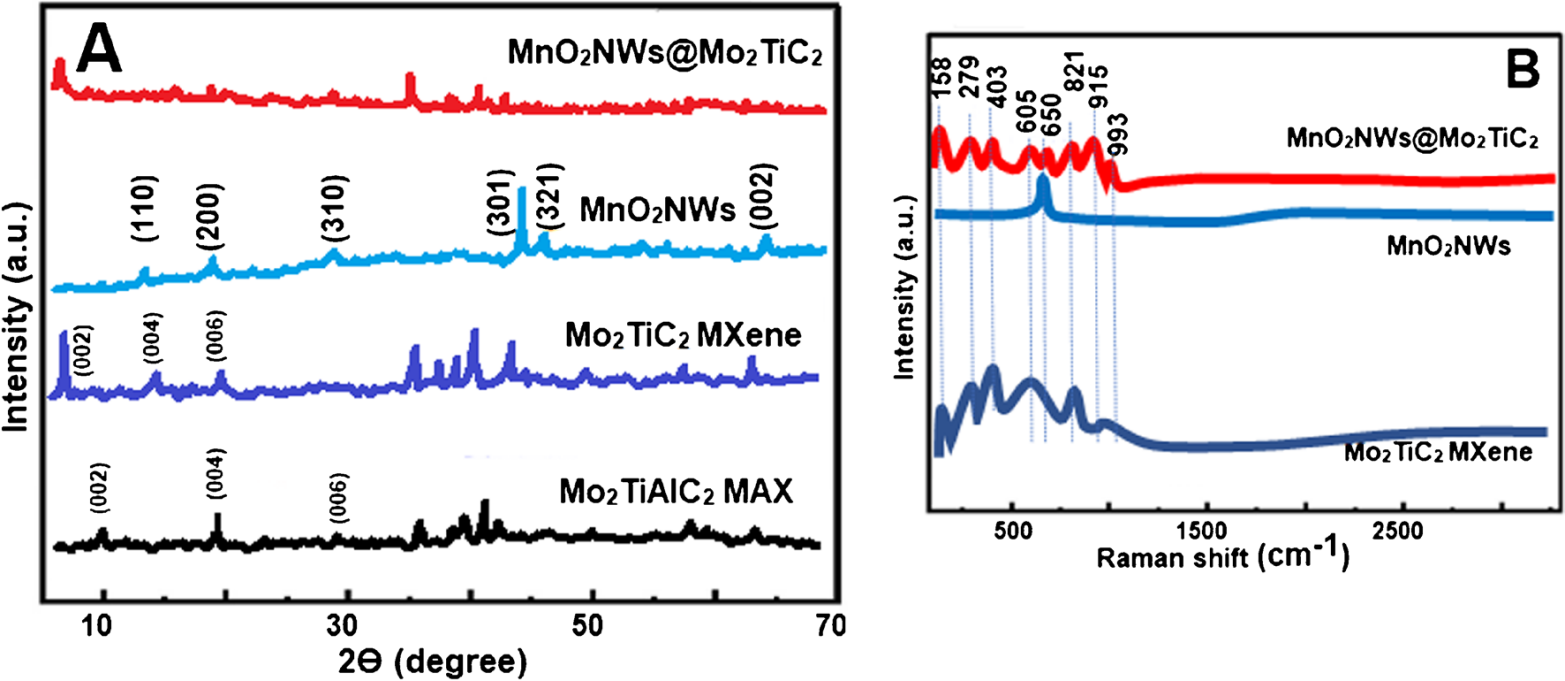
**A** XRD pattern and **B** Raman spectra of MnO_2_NWs@Mo_2_TiC_2_ nanocomposite, Mo_2_TiAlC_2_ MAX, Mo_2_TiC_2_ MXene and MnO_2_NWs

To highlight the structural features of the synthesized nanomaterials including MnO_2_NWs@Mo_2_TiC_2_ nanocomposite, Mo_2_TiAlC_2_ MAX, Mo_2_TiC_2_ MXene and MnO_2_NWs, XRD measurements were firstly performed (Fig. 1A). According to XRD pattern of MAX phase, the peaks at 9.27°, 19.54° and 28.93° corresponded to (002), (004) and (006) planes, respectively. After the preparation of MXene structure, XRD peaks belonging to MAX phase were shifted to lower angles at 6.37°, 14.53° and 18.07° attributing to (002), (004) and (006) planes, respectively [30, 32]. The shifts to lower XRD angles indicated that the parameters including d-spacing and c-lattice increased owing to H_2_O molecule’s intercalation and the surface termination attachment to MXene sheets replacing A element [33]. The more intensity peak corresponding to MXene (002) plane in comparison with MAX phase suggested that the aluminum etching and the attachment of surface functional groups increased structural stability and MXene crystallinity. Moreover, XRD peaks between 35° and 45° showed the presence of unreacted MAX phase in MXene structure. In addition, according to XRD peaks of MnO_2_NWs at 12.36°, 18.07°, 29.04°, 45.27° and 64.09° attributing to (110), (200), (310), (301), (321) and (002) planes, respectively, the presence of MnO_2_NWs was confirmed [30]. The XRD pattern of MnO_2_NWs@Mo_2_TiC_2_ nanocomposite verified the successful combination between MnO_2_NWs and Mo_2_TiC_2_ MXene. In addition, XRD peak belonging to the nanocomposite at about 5.45° attributing to (002) plane was shifted to the lower angle degree in comparison with Mo_2_TiAlC_2_ MAX and Mo_2_TiC_2_ MXene, and thus, this situation confirmed the successful intercalation between MnO_2_NWs and Mo_2_TiC_2_ MXene.

Raman spectra of MnO_2_NWs@Mo_2_TiC_2_ nanocomposite, Mo_2_TiC_2_ MXene and MnO_2_NWs were recorded (Fig. 1B). Raman absorption bands at 158, 279, 403, 605, 821, 915 and 993 cm^−1^ were observed in Raman spectra of Mo_2_TiC_2_ MXene, and these bands confirmed the presence of Mo_2_TiC_2_ MXene in harmony with literature [32, 34]. Raman band at 158 cm^−1^ was in harmony with *E*_*g*_ in-plane vibration due to the presence of molybdenum and titanium elements in MXene structure and Raman band at 279 cm^−1^ corresponded to *E*_*g*_ mode vibration owing to the oxygen element. In addition, Raman bands at 403 and 605 cm^−1^ were emerged because of carbon vibrations in MXene structure [35]. Moreover, Raman bands at 821, 915 and 993 cm^−1^ were attributed to distinct Raman modes owing to the functionalized oxygen elements in MXene structure [36]. Finally, Raman band at 650 cm^−1^ belonging to MnO_2_NWs indicated the Mn–O-stretching vibration, providing the presence of MnO_2_NWs in MXene [37].

The thermogravimetric (TGA) plots (Fig. S1) were recorded for MnO_2_NWs@Mo_2_TiC_2_ nanocomposite, Mo_2_TiC_2_ MXene and MnO_2_NWs in N_2_ gas presence until 1000°C. Firstly, according to TGA plot of MXene structure, the plot was almost constant between 25 and 1000°C and minor weight loss (about 1.0%) was only observed between 25 and 1000°C owing to H_2_O molecule’s adsorption and the removal of surface functional groups in MXene structure [38]. Due to the strong bands including Mo–C and Ti–C in MXene structure, the thermal stability of MXene was observed at even elevated temperatures. According to TGA plot of MnO_2_NWs, the weight loss (about 3.0%) occurred up to 1000°C because of the moisture removal and the phase conversion of MnO_2_ into Mn_2_O_3_ [39]. The weight losses occurred in three stages on TGA plot of MnO_2_NWs@Mo_2_TiC_2_ nanocomposite owing to the moisture removal (1.8%) between 100 and 200°C, the removal of surface terminal groups in MXene structure and the phase conversion of MnO_2_ into Mn_2_O_3_ (about 4.0%) between 250 and 350°C. Finally, the weight loss (about 6.0%) occurred between 550 and 700°C owing to the modifier’s decomposition on MXene surface after the washing treatment.

FTIR spectra (Fig. S2) was obtained for MnO_2_NWs@Mo_2_TiC_2_ nanocomposite, Mo_2_TiC_2_ MXene and MnO_2_NWs. The absorption peaks at 3431 cm^−1^ belonging to O–H stretching, 2930 and 2841 cm^−1^ belonging to C–H stretching, 1629 cm^−1^ showing O–H bending and 1157 and 1068 cm^−1^ demonstrating C–O stretching were observed in the whole nanomaterials [40]. In addition, the absorption bands of Mo_2_TiC_2−_MXene were observed at 580 and 503 cm^−1^ owing to Mo–O and Ti–O groups’ stretching and the absorption band at 588 cm^−1^ showed Mn–O vibration in MnO_2_NWs [41]. Finally, the absorption band between 580 and 500 cm^−1^ showed the vibration modes corresponding to M–O or M–O–M (M=Mo, Ti and Mn) stretching, confirming the successful preparation of MnO_2_NWs@Mo_2_TiC_2_ nanocomposite.

BET measurements (Fig. S3) were carried out to calculate the specific surface areas of MnO_2_NWs@Mo_2_TiC_2_ nanocomposite, Mo_2_TiC_2_ MXene and MnO_2_NWs owing to their significant role in electrochemical activity and sensor applications. The specific surface areas of Mo_2_TiC_2_ MXene, MnO_2_NWs and MnO_2_NWs@Mo_2_TiC_2_ nanocomposite were determined as 15.31±0.07 m^2^ g^−1^, 18.13±0.02 m^2^ g^−1^ and 107.84±0.04 m^2^ g^−1^, respectively. Thus, MnO_2_NWs@Mo_2_TiC_2_ nanocomposite demonstrated the highest specific surface area due to one-dimensional and two-dimensional structure interactions. The pore-size distribution was investigated by using the non-local density functional theory for Mo_2_TiC_2_ MXene, MnO_2_NWs and MnO_2_NWs@Mo_2_TiC_2_ nanocomposite, confirming the harmony with BET measurements.

**Fig. 2:**
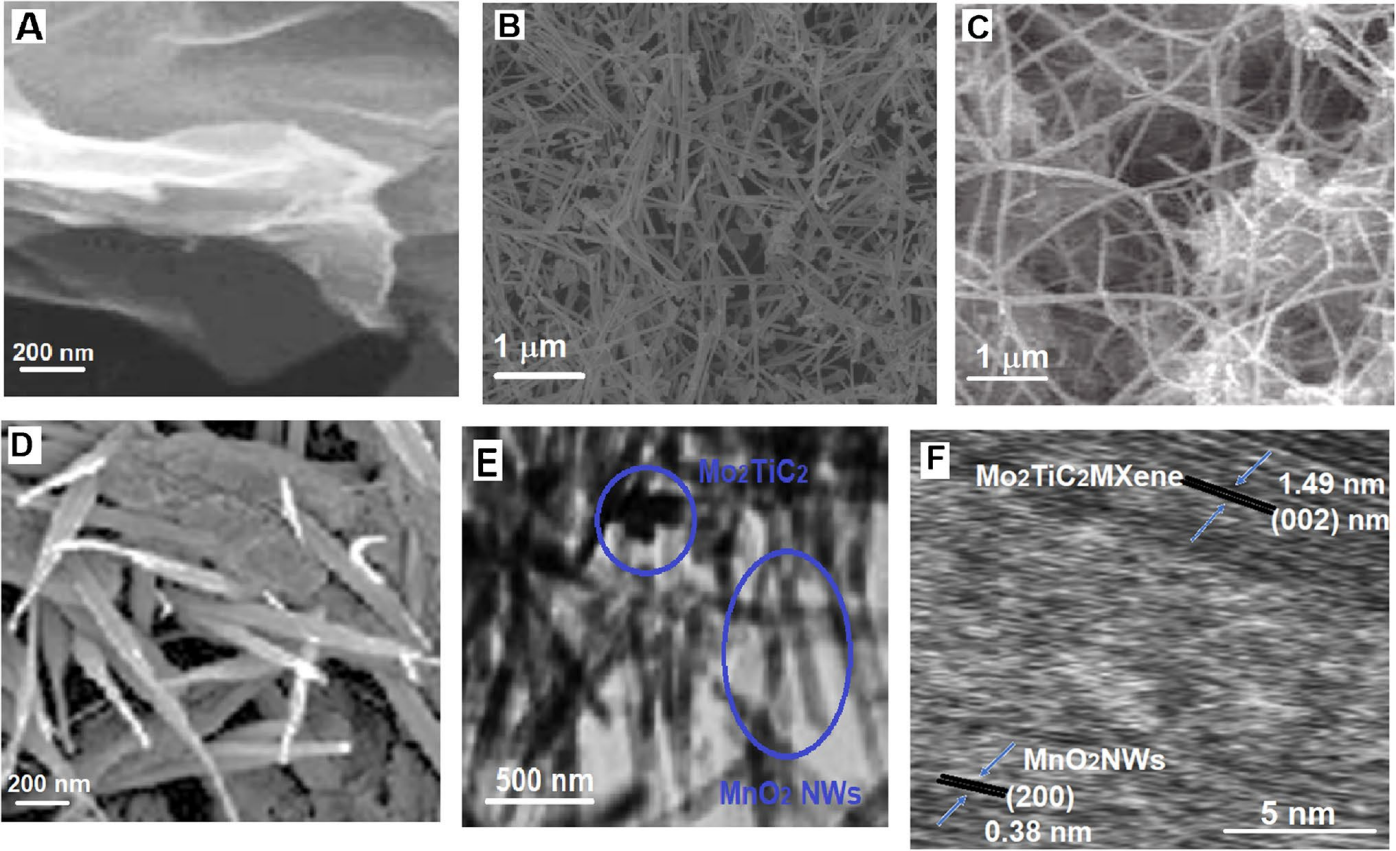
FESEM images of **A** Mo_2_TiC_2_ MXene, **B** MnO_2_NWs, **C** and **D** MnO_2_NWs@Mo_2_TiC_2_ nanocomposite, TEM image **E** of MnO_2_NWs@Mo_2_TiC_2_ nanocomposite and HRTEM image **F** of MnO_2_NWs@Mo_2_TiC_2_ nanocomposite

FESEM images (Fig. 2) were recorded to study the surface morphologies of MnO_2_NWs@Mo_2_TiC_2_ nanocomposite, Mo_2_TiC_2_ MXene and MnO_2_NWs. According to Fig. 2A, MXene structure flakes were observed to provide the interaction with MnO_2_NWs forming the nanocomposite. Figure 2B showed MnO_2_NWs’ surface morphology providing the effective intercalation with MXene structure. According to Fig. 2C and Fig 2D, MnO_2_NWs was pillared between the pillared CTAB MXene flakes and the uniform distribution of MnO_2_NWs was observed on MXene surface. Thus, this composite allows improving the electrochemical performance of transition metal oxides by avoiding the restacking problem among the MXene sheets. According to Fig. 2E belonging to MnO_2_NWs@Mo_2_TiC_2_ nanocomposite, the perfect interaction of Mo_2_TiC_2_ MXene with ultra-thin MnO_2_NWs was observed. Finally, d-spacing values including 1.49 nm of Mo_2_TiC_2_ MXene for (002) phase and 0.38 nm of MnO_2_NWs for (200) phase were given in Fig. 2F. Hence, FESEM, TEM and HRTEM images showed the successful preparation of MnO_2_NWs@Mo_2_TiC_2_ nanocomposite. In addition, EDX spectra of Mo_2_TiC_2_ MXene, MnO_2_NWs and MnO_2_NWs@Mo_2_TiC_2_ nanocomposite was given Fig. S4, and these results verified the successful synthesis of MnO_2_NWs@Mo_2_TiC_2_ nanocomposite [30].

**Fig. 3:**
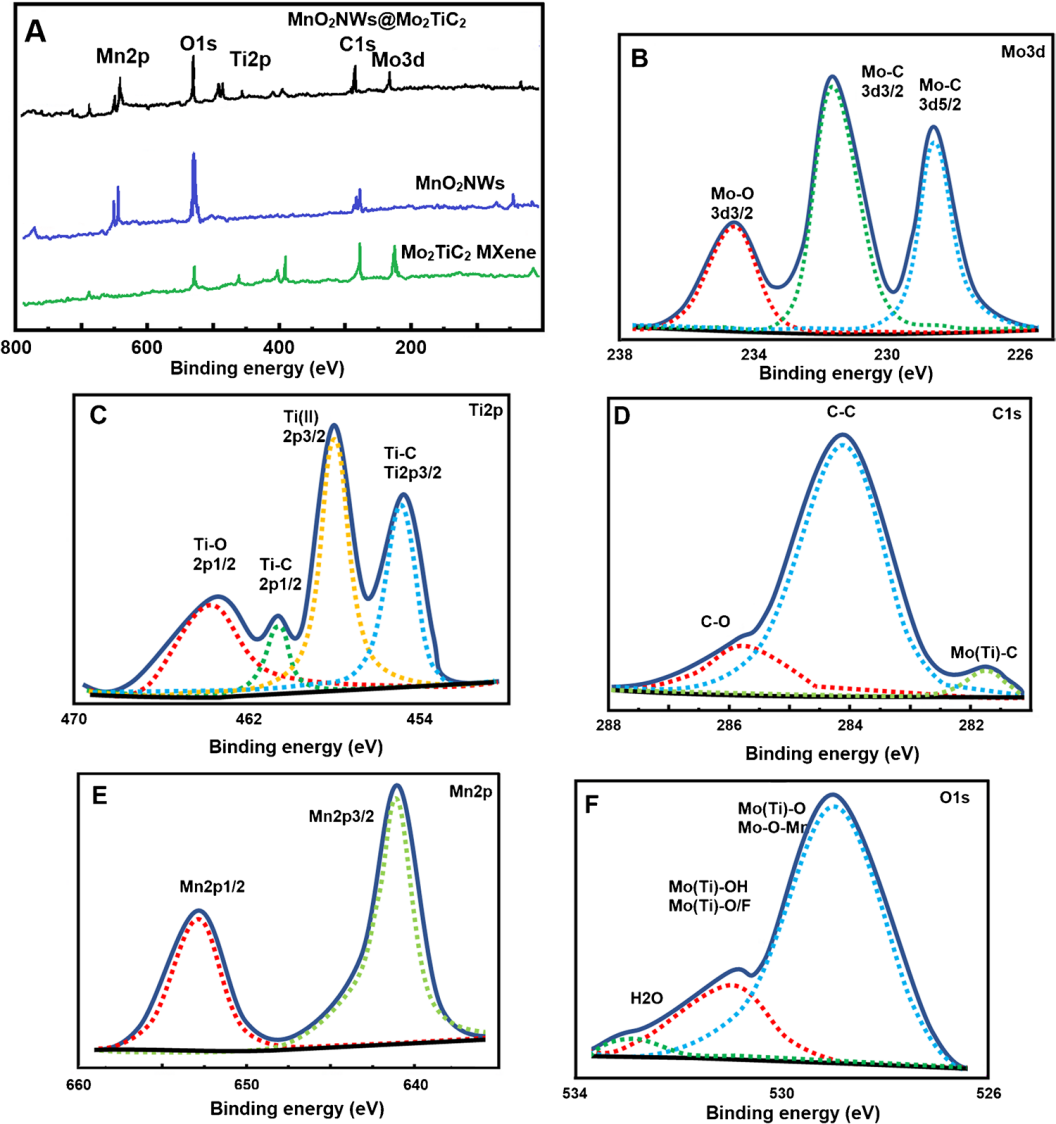
Survey spectra of **A** Mo_2_TiC_2_ MXene, MnO_2_NWs and MnO_2_NWs@Mo_2_TiC_2_ nanocomposite and high-resolution XPS spectra of MnO_2_NWs@Mo_2_TiC_2_ nanocomposite **B** Mo3d, **C** Ti2p, **D** C1s, **E** Mn2p and **F** O1s

Survey spectra of Mo_2_TiC_2_ MXene, MnO_2_NWs and MnO_2_NWs@Mo_2_TiC_2_ nanocomposite and high-resolution XPS spectra of Mo3d, Ti2p, C1s, Mn2p and O1s were given on Fig. 3. According to the survey spectra of Mo_2_TiC_2_ MXene, MnO_2_NWs and MnO_2_NWs@Mo_2_TiC_2_ nanocomposite (Fig. 3A), the presence of molybdenum, titanium, carbon, manganese and oxygen verified the successful production of Mo_2_TiC_2_ MXene, MnO_2_NWs and MnO_2_NWs@Mo_2_TiC_2_ nanocomposite [30]. According to Mo3d XPS spectrum (Fig. 3B), three XPS peaks Mo–O (3d3/2), Mo–C (3d3/2) and Mo–C (3d5/2) at 233.89, 232.13 and 229.31 eV were observed, respectively [42]. XPS peaks relating to Ti–O (2p1/2), Ti–C (2p1/2), Ti (II) (2p3/2) and Ti–C (2p3/2) were observed at 463.87, 461.18, 458.21 and 455.36 eV, respectively for Ti2p (Fig. 3C) [32]. XPS peaks attributing to C1s corresponded to C–O, C–C and Mo (Ti)–C bonds at 286.37, 285.03 and 282.27 eV, respectively (Fig. 3D) [43]. Moreover, two XRD peaks attributing to Mn2p corresponded to Mn2p1/2 and Mn2p3/2 at 653.13 and 640.89 eV, respectively (Fig. 3E). Finally, according to O1s XPS spectrum (Fig. 3F), XPS peaks corresponding to H_2_O, Mo (Ti)–O–OH, Mo (Ti)–O–O/F, Mo (Ti)–O and Mn–O–Mn were observed at 533.12, 531.21 and 529.31 eV, respectively.

### Electrochemical works of Mo_2_TiC_2_ MXene, MnO_2_NWs and MnO_2_NWs@Mo_2_TiC_2_-modified electrodes

**Fig. 4:**
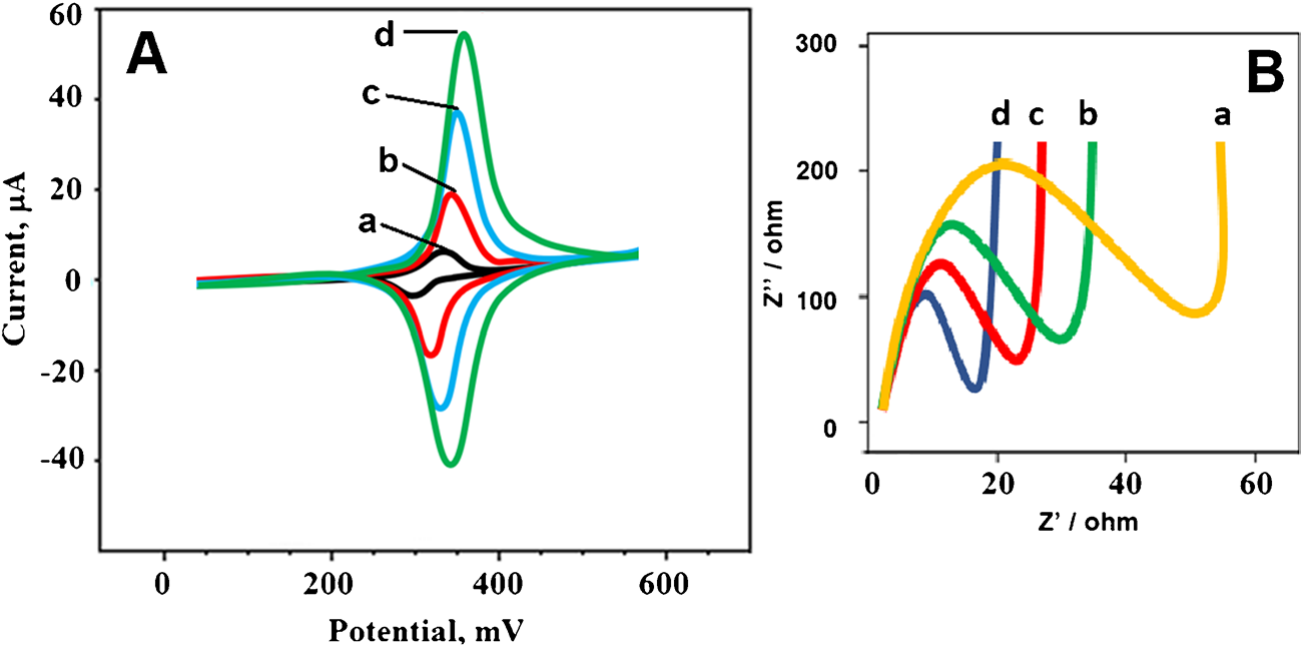
**A** CV curves and **B** EIS responses at (a) bare GCE, (b) Mo_2_TiC_2_ MXene/GCE, (c) MnO_2_NWs/GCE, (d) MnO_2_NWs@Mo_2_TiC_2_/GCE (redox probe: 1.0 mmol L^−1^ [Fe(CN)_6_]^3−/4−^ containing 0.1 mol L^-1^ KCl, potential scan rate: 50 mV s^-1^)

Electrochemical characterization was carried out using EIS and CV techniques for the comparisons of the prepared electrode materials such as Mo_2_TiC_2_ MXene, MnO_2_NWs and MnO_2_NWs@Mo_2_TiC_2_ (Fig. 4A). The observed anodic and cathodic peaks using bare GCE (curve a) had smaller current values than that of using Mo_2_TiC_2_ MXene/GCE at (curve b) owing to MXene structure’s superior electrical conductivity, the conductive channels providing easy charge mobility and specific surface area [13, 44]. When the obtained electrochemical activity values using Mo_2_TiC_2_ MXene/GCE and MnO_2_NWs/GCE electrodes were compared, it was expected that the obtained anodic/cathodic current values using MnO_2_NWs/GCE were higher due to the larger specific surface area of MnO_2_NWs (curve c). The electroactive surface areas of bare GCE, Mo_2_TiC_2_ MXene/GCE, MnO_2_NWs/GCE and MnO_2_NWs@Mo_2_TiC_2_/GCE were calculated as 0.070±0.004, 0.237±0.001, 0.438±0.005 and 1.619±0.007 cm^2^ by using *i*_*p*_ = 2.69×10^5^ A n^3/2^ D^1/2^ C v^1/2^ equation in presence of 1.0 mmol L^−1^ [Fe(CN)_6_]^3−^, respectively. According to these CV results, the easy restacking of MXene sheets with ultrathin MnO_2_NWs in presence of CTAB and 1D/2D intertwined heterostructure of the nanocomposite provided the efficient synergistic effects between MnO_2_NWs and Mo_2_TiC_2_MXene and the highest electrochemical activity was observed on MnO_2_NWs@Mo_2_TiC_2_/GCE (curve d).

Moreover, EIS measurements (Fig. 4B) were carried out to compare the electrode conductivities. The charge transfer resistance (*R*_ct_) values were conducted to be 50 ohm for bare GCE (curve a), 30 ohm for Mo_2_TiC_2_ MXene/GCE (curve b), 20 ohm for MnO_2_NWs/GCE (curve c) and 15 ohm for MnO_2_NWs@Mo_2_TiC_2_/GCE (curve d), offering the harmony results in CV and EIS results. Lastly, some electrochemical investigations were completed by the evaluations of the electron transfer rate constant (*k*^0^) and the electrochemical reaction type. *k*^0^ was calculated as 1.61×10^−3^ cm s^−1^ by using the equation of *k*^*0*^* = 2.415 exp (−0.02F/RT) D*^*1/2*^*(E*_*p*_* – E*_*p/2*_*)*^*−1/2*^* v*^*1/2*^ at 25°C, confirming irreversible electrochemical mechanism on MnO_2_NWs@Mo_2_TiC_2_/GCE [45]. Then, the slope of *i*_*p*_* = −2.99×10*^*5*^* n (αn*_*a*_*)*^*1/2*^* C D*^*1/2*^* v*^*1/2*^ equation at 25°C (*n*_*a*_ and *n* mean the number of electrons in the rate-determining step and the total number of electrons, respectively; *α* means the charge transfer coefficient; C/mol cm^−3^ means FEN concentration; D/cm^2^ s^−1^ means the diffusion coefficient and v/s^−1^ means scan rate) was obtained as 0.47, suggesting the diffusion controlled electrochemical reaction [46].

### Development of FEN imprinted polymer on MnO_2_NWs@Mo_2_TiC_2_/GCE

Fig. S5A showed the electro-polymerization voltammogram in presence of 100.0 mmol L^−1^ Py monomer and 25.0 mmol L^−1^ FEN target molecule on MnO_2_NWs@Mo_2_TiC_2_/GCE. Since the applied high potential especially in electro-polymerization techniques significantly affected the stability of the complex to be formed between monomer and target molecule, a potential between +0.0/+1.0 V was applied in this study. As a result of the first scan, the anodic peak current, which appeared at a high intensity at approximately +0.70 V, started to decrease with the number of scans and eventually approached almost zero at the 25^th^ scan number. This situation demonstrated the formation of analyte molecule imprinting polymer layers on MnO_2_NWs@Mo_2_TiC_2_/GCE.

To confirm the high selectivity of MIP/MnO_2_NWs@Mo_2_TiC_2_/GCE in comparison with NIP/MnO_2_NWs@Mo_2_TiC_2_/GCE, these two electrodes were used in the presence of 10.0 nmol L^−1^ FEN, and it was seen that the FEN peak current obtained when MIP electrode was used is approximately ten times the FEN peak current obtained when NIP electrode was used (Fig. S5B). In addition, the small FEN currents obtained using NIP electrodes occurred due to nonspecific interactions on the electrode surface. These results showed that the obtained imprinting selectivity using the electro-polymerization technique was quite good.

Finally, four FEN imprinting electrodes such as MIP/bare GCE, MIP/Mo_2_TiC_2_ MXene/GCE, MIP/MnO_2_NWs/GCE, and MIP/MnO_2_NWs@Mo_2_TiC_2_/GCE were developed and applied in presence of 10.0 nmol L^−1^ FEN. According to Fig. S5C, as expected, the highest peak current was obtained using MIP/MnO_2_NWs@Mo_2_TiC_2_/GCE in harmony with CV and EIS measurements.

### Optimization

#### pH effect

**Fig. 5:**
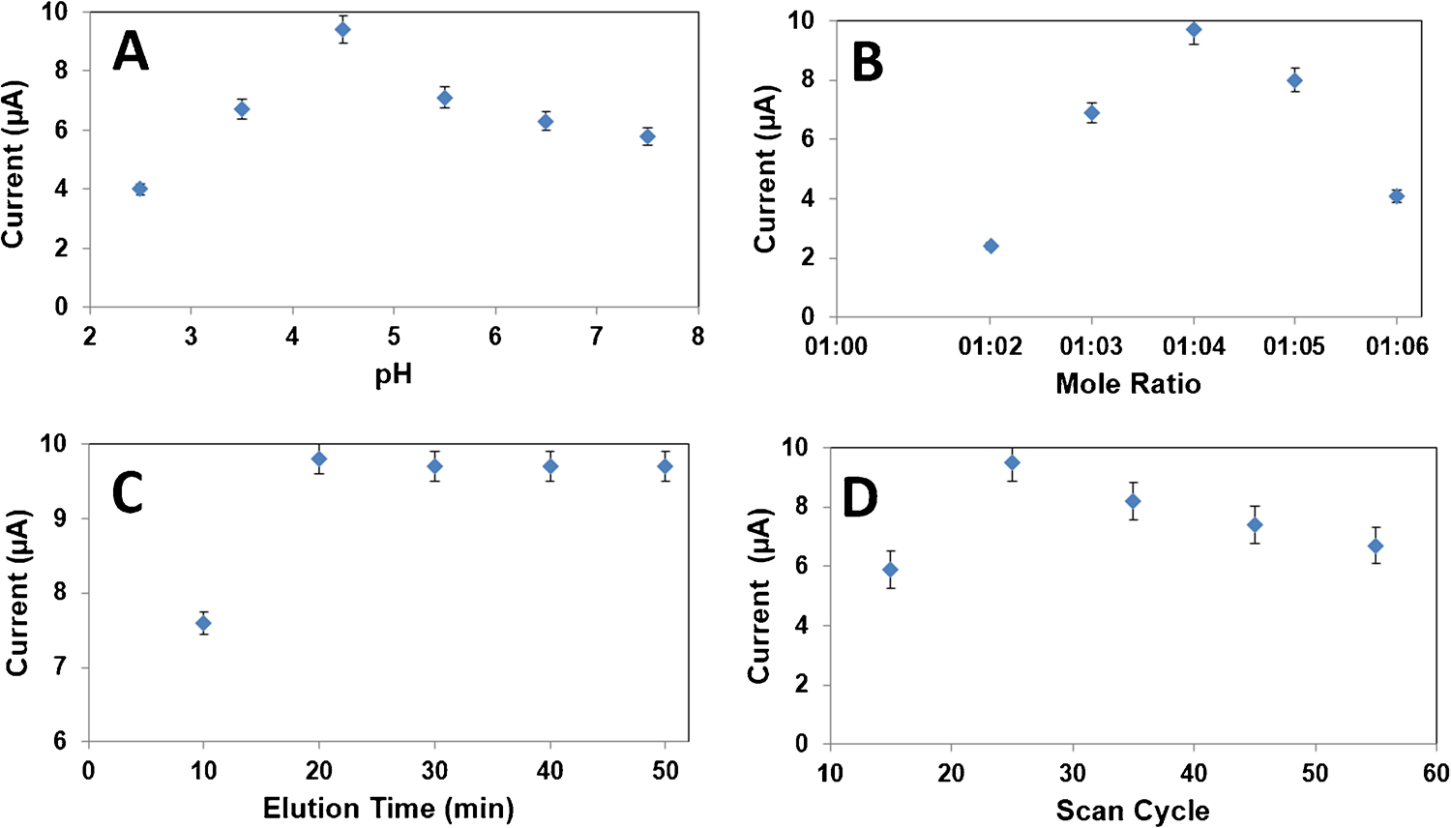
Effect of **A** pH, **B** mole ratio, **C** desorption time, **D** scan cycle on signals of SWVs (in presence of 10.0 nmol L^−1^ FEN) (*n* = 6)

In electrochemical studies, the supporting electrolyte pH is the most important factor affecting the sensitivity and applicability of the developed sensor. For this purpose, significant studies were carried out in the pH range of 2.5–7.5 to see the effect of the supporting electrolyte pH on the FEN peak current. According to Fig. 5A, highest reduction signal belonging to FEN was observed at pH 4.5. The proton amount in slightly weakly acidic supporting electrolyte was used for nitro group’s reduction corresponding to FEN determination [47]. In addition, the reduction potentials were linear with the supporting electrolyte pH with a slope value of −42.87 mV/pH, suggesting 1.0 of the ratio of protons to electrons number. Thus, the reduction of NO_2_ group to −NH(OH) occurred on MIP/MnO_2_NWs@Mo_2_TiC_2_/GCE (Fig. S6) [48].

#### Mole ratio FEN to Py monomer effect

In MIP-based electrochemical sensor applications, the stability of the complex that forms between the target molecule and the monomer is an important factor. While a stable complex does not form when the monomer ratio is kept low, it is difficult to remove the target molecule from the electrode surface when the monomer ratio is high. Thus, FEN imprinting polymer on MnO_2_NWs@Mo_2_TiC_2_/GCE was prepared by using 100.0 mmol L^−1^ Py monomer and 25.0 mmol L^−1^ target molecule in this study, providing the highest electrochemical signals (Fig. 5B).

#### Desorption time effect

In order to obtain a high sensor signal in MIP-based electrochemical sensor applications, the target molecule deposited on the electrode surface must be almost completely removed from the electrode surface. In this study, desorption times between 10 and 50 min were applied, and after the 20^th^ min, it was observed that the FEN molecules were almost completely removed from the electrode surface and maximum electrochemical signals were obtained (Fig. 5C).

#### Scan cycle effect

In electro-polymerization techniques, the polymer thickness obtained on the electrode surface using monomer and target molecule affects the method sensitivity. Since the polymer thickness on the electrode surface can be thin at low scanning numbers, the ruptures may occur on the electrode surface. On the contrary, the thick polymer layer that forms on the electrode surface at high scanning numbers, can cause non-specific interactions, which may negatively affect the sensor performance. Thus, according to Fig. 5D, the optimal scan cycle number was selected as 25.

### Sensitivity of MIP/MnO_2_NWs@Mo_2_TiC_2_/GCE sensor

**Fig. 6:**
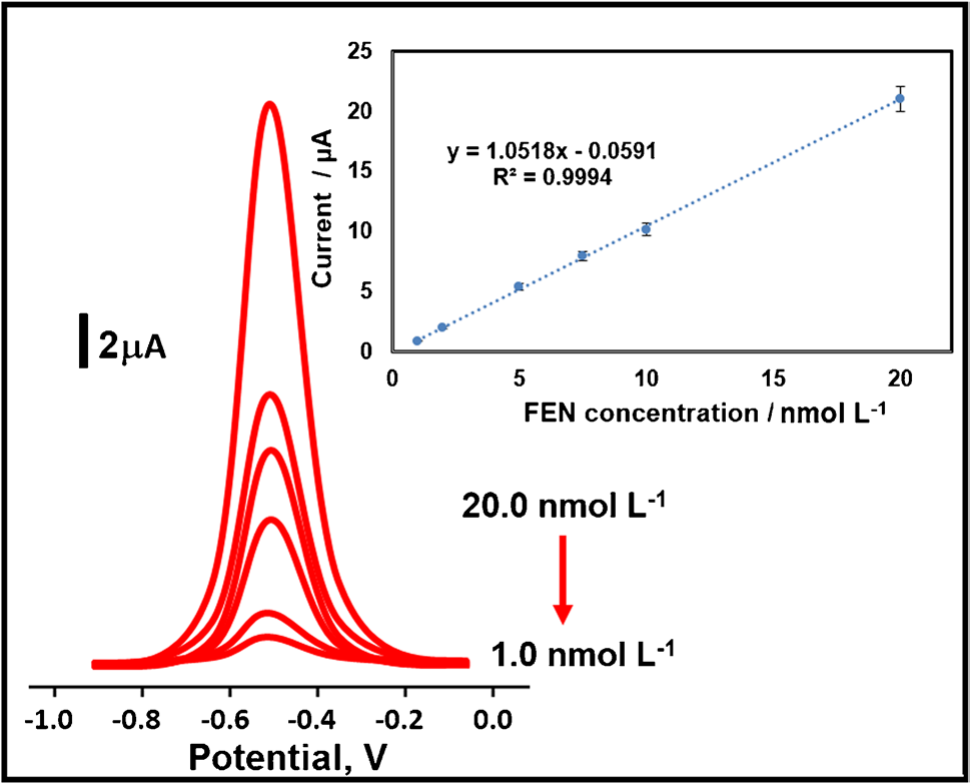
FEN concentration effect on MIP/MnO_2_NWs@Mo_2_TiC_2_/GCE in presence of pH 4.5 of PBS (from 1.0×10^−9^ to 2.0×10^−8^ mol L^−1^ FEN) by using SWV method. Inset: Calibration curve of FEN concentrations against the obtained peak currents

Calibration equation of *y* (µA) = 1.0518x (*C*_FEN_, nmol L^−1^)–0.0591, (*R*^2^ = 0.9994) was obtained using the obtained peak current values by applying the developed MIP-based electrode to the prepared standard FEN solutions including various concentrations (Fig. 6). The values of LOQ and LOD were calculated as 1.0×10^−9^ mol L^−1^ and 3.0×10^−10^ mol L^−1^, respectively (see Supplementary Data for the equations). According to this LOD value (Table 1), it is possible to say that a MIP-based electrochemical sensor with higher sensitivity for FEN detection has been successfully prepared in this study in comparison with the other methods owing to MXenes’ superior physical properties including high conductivity and low diffusion barriers and MnO_2_’s high-capacitive performance. Especially, the increase in pesticide usage and their unconscious consumption in recent years cause pesticide-induced metabolic disorders and serious diseases. Hence, the early diagnosis of such diseases is possible thanks to this developed MIP/MnO_2_NWs@Mo_2_TiC_2_/GCE sensor. Moreover, during the preparation of the MIP-based sensor, a little waste generation was observed as a result of the synthesis of MnO_2_NWs nanomaterial by a facile hydrothermal method and the synthesis of the nanocomposite material by the pillaring method. As a result, it was possible to say that the prepared sensor was environmentally and human friendly in accordance with green chemistry. In addition, an inexpensive sensor with a fast response time has been presented to the scientific literature for the miniaturization because CV electro-polymerization technique was used during the sensor preparation and the performance applications were performed with SWV technique as a fast voltametric technique. In comparison with FEN detection methods based on enzymatic materials, these enzymatic sensors demonstrated low stability and mild conditions in the laboratory unlike the conditions in this study [49].

**Table 1:** Comparison of the prepared MIP sensor with the other novel analytical methods

Material	Linear range (mol L^−1^)	LOD (mol L^−1^)	Reference
GCp/g-C_3_N_4_@Co-doped CeO_2_	1.0×10^−8^–13.7×10^−6^	3.2×10^−9^	[50]
Spinel Ni–Fe–O	1.0×10^−8^–1.0×10^−6^	1.0×10^−8^	[51]
Ni,N-CDs/Fe_3_O_4_@ZIF-8	9.0×10^−10^–1.2×10^−7^	8.0×10^−10^	[52]
Poly (safranine)	1.0×10^−6^–1.5×10^−5^	1.0×10^−7^	[53]
NH_2_-MIL-125(Ti)/reduced graphene oxide	7.2×10^−8^–1.8×10^−5^	3.4×10^−8^	[54]
Poly-arginine/graphene oxide	6.9×10^−7^–2.0×10^−5^	1.73×10^−7^	[55]
Dysprosium vanadate 3D-micro flowers	1.0×10^−7^–1.4×10^−4^	1.4×10^−9^	[56]
Tyrosinase/poly(2-hydroxybenzamide)	1.8×10^−8^–3.6×10^−6^	4.7×10^−9^	[57]
MIP/MnO_2_NWs@Mo_2_TiC_2_	1.0×10^−9^–2.0×10^−8^	3.0×10^−10^	This study

### Recovery

To demonstrate the usability of the prepared MIP based sensor in real food samples (white flour), it was applied to real samples and recovery values were calculated by using standard addition method (Table S1). Before proceeding with the analyses, the white flour sample, which was prepared for analysis in section of sample preparation, was divided into four equal conical flasks, and standard solutions of increasing FEN concentrations (2.00, 4.00 and 6.00 nmol L^−1^) were added to the other flasks except the first conical flask. FEN analysis was performed in these four samples thanks to MIP/MnO_2_NWs@Mo_2_TiC_2_/GCE sensor. The values close to 100% proved that the prepared sensor could recognize and determine FEN with high selectivity. In addition, the obtained calibration equation using the above standard addition method was extracted as *y* (µA) = 1.0495x (*C*_FEN_, nmol L^−1^) + 0.1893, (*R*^2^ = 0.9995). The fact that the slopes of the obtained calibration equations by the direct calibration method and the standard addition method were close to each other showed that FEN analysis could be performed with high selectivity on real white flour samples.

### Selectivity, stability and reproducibility of MIP/MnO_2_NWs@Mo_2_TiC_2_

To show the high sensor selectivity of MIP/MnO_2_NWs@Mo_2_TiC_2_/GCE, the selectivity test was planned in presence of FEN, and the other highly interfering agents such as METP, MAL, VIN and HQ in 0.1 M PBS (pH 4.5). According to Fig. S7 and Table S2, the prepared sensor based on MIP and MnO_2_NWs@Mo_2_TiC_2_ nanocomposite showed the high affinity towards FEN in presence of other highly interfering agents. Finally, the values of the selectivity coefficient (*k*) and relative selectivity coefficient (*k*') confirmed the high affinity of molecularly imprinting technology towards target molecule in real samples.

Secondly, the stability investigation of only one MIP/MnO_2_NWs@Mo_2_TiC_2_/GCE sensor was conducted. For this aim, the square wave voltammograms against 10.0 nmol L^−1^ FEN were obtained by using one MIP/MnO_2_NWs@Mo_2_TiC_2_/GCE sensor during 7 weeks and the observed Δ*i* (µA) value at the end of the seventh week was approximately 96.73% of the observed Δ*i* (µA) value at the end of the first week, confirming the high stability (Fig. S8).

For the reproducibility test of MIP/MnO_2_NWs@Mo_2_TiC_2_/GCE, 20 different MIP-based electrochemical electrodes were prepared according to the procedure detailed in ‘Preparation of MnO2NWs@Mo2TiC2 modified glassy carbon electrode (MnO2NWs@Mo2TiC2 /GCE)’ and Sample preparation’. The obtained average peak currents using these 20 MIP different electrodes were calculated in presence of 10.0 nmol L^−1^ FEN by using SWV method and the relative standard deviation was found to be 0.63%, offering a high reproducibility.

## Conclusions

In present research, the cyclic voltammetric electro-polymerization of pyrrole monomer solution on MnO_2_NWs@Mo_2_TiC_2_ nanocomposite-modified glassy carbon electrode was successfully designed and used for the direct detection of the toxic pesticide fenitrothion. The unique aspect of this study was that this type of MIP and nanocomposite-based sensor was developed for the first time and used for the analysis of fenitrothion from real samples. The prepared electrode surfaces and synthesized nanomaterials were characterized with electroanalytical, spectroscopic and microscopic methods. According to the characterization results, MnO_2_NWs@Mo_2_TiC_2_ nanocomposite showed good conductivity, large surface area and strong sensor interface for fenitrothion detection. The developed MIP sensor indicated a linear range from 1.0×10^−9^ to 2.0×10^−8^ mol L^−1^ with a low LOD (3.0×10^−10^ mol L^−1^). The high selectivity of the developed sensor was proved in presence of other highly interfering agents and the sensor was utilized for the sensitive determination of fenitrothion in white flour samples with the satisfactory results, providing the practical application. The sensor was also inexpensive, environmentally and human friendly while the preparation processes of the sensor and the nanocomposite synthesis, ensuring easy-to fabrication type of an electrochemical sensor. Thus, the prepared molecularly imprinting sensor can be a promising analytical device for the other pesticides determination in food analysis processes.

### Supplementary Information

Below is the link to the electronic supplementary material.

Supplementary file1 (DOC 506 KB)

## Data Availability

Data will be made available on request.
