# Supplementary material for: Electrochemical determination of fenitrothion pesticide based on ultrathin manganese oxide nanowires/molybdenum titanium carbide MXene ionic nanocomposite and molecularly imprinting polymer
Source: Mikrochim Acta. 2024 Apr 2;191(5):230. doi: 10.1007/s00604-024-06320-5 (PMC10987362; doi:10.1007/s00604-024-06320-5)
Supplement: Supplementary file 1 — Supplementary file1 (DOC 506 KB) [file 604_2024_6320_MOESM1_ESM.doc]

**Supplementary Data**

**For**

**Electrochemical determination of fenitrothion pesticide based on ultrathin manganese oxide nanowires/molybdenum titanium carbide MXene ionic nanocomposite and molecularly imprinting polymer**

Bahar Bankoğlu Yola^1^, Gül Kotan^2^, Onur Akyıldırım^3^, Necip Atar^4^, Mehmet Lütfi Yola^5*^

*^1^Department of Engineering Basic Sciences, Faculty of Engineering and Natural Sciences, Gaziantep Islam Science and Technology University, Gaziantep, Turkey*

*^2^Department of Chemistry and Chemical Processing Technologies, Kars Vocational School, Kafkas University, Kars, Turkey*

*^3^Department of Chemical Engineering, Faculty of Engineering and Architecture, Kafkas University, Kars, Turkey*

*^4^Department of Chemical Engineering, Faculty of Engineering, Pamukkale University, Denizli, Turkey*

*^5^Department of Nutrition and Dietetics, Faculty of Health Sciences, Hasan Kalyoncu University, Gaziantep, Turkey*

**Correspondence: mlutfi.yola@hku.edu.tr; Tel.: +90-3422118080; Fax: +90-3422118081*

**Instrumentation**

Some analytical devices for the morphological analyzes such as ZEISS EVO 50 SEM (Tokyo, Japan) for field emission scanning electron microscopy (FESEM), JEOL 2100 TEM/HRTEM instrument for transmission electron microscopy (TEM), Rikagu Miniflex, x-ray diffractometer (Tokyo, Japan) for x-ray diffraction analysis (XRD), Bruker-Tensor 27 FTIR spectrometer (Tokyo, Japan) for fourier-transform infrared spectroscopy (FTIR) and PHI 5000 Versa Probe type x-ray photoelectron spectrometer (Japan/USA) for x-ray photoelectron spectroscopy (XPS) were applied in present study.

**Sensitivity**

*LOQ = 10.0 S / m*

*LOD = 3.3 S / m*

S: Standard deviation of the intercept and m*:* Slope of the regression line

**
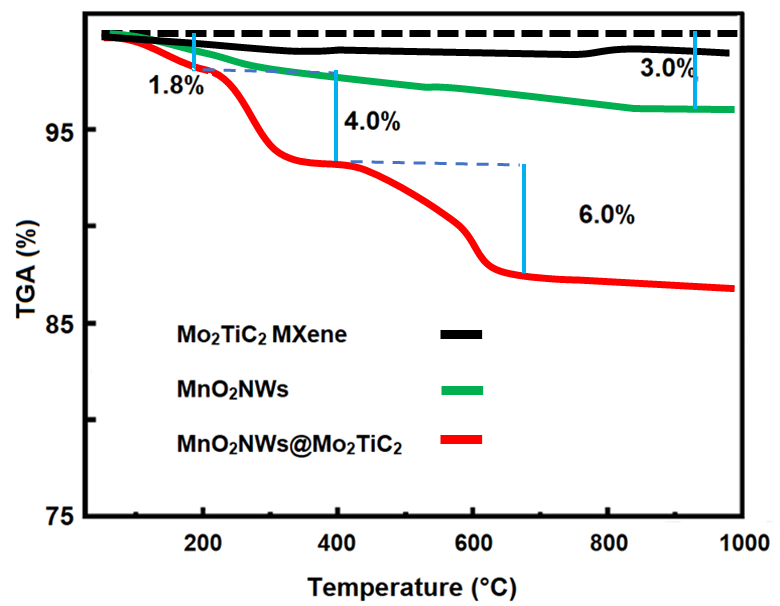
Fig. S1** TGA plot of MnO_2_NWs@Mo_2_TiC_2_ nanocomposite, Mo_2_TiC_2_ MXene and MnO_2_NWs

**
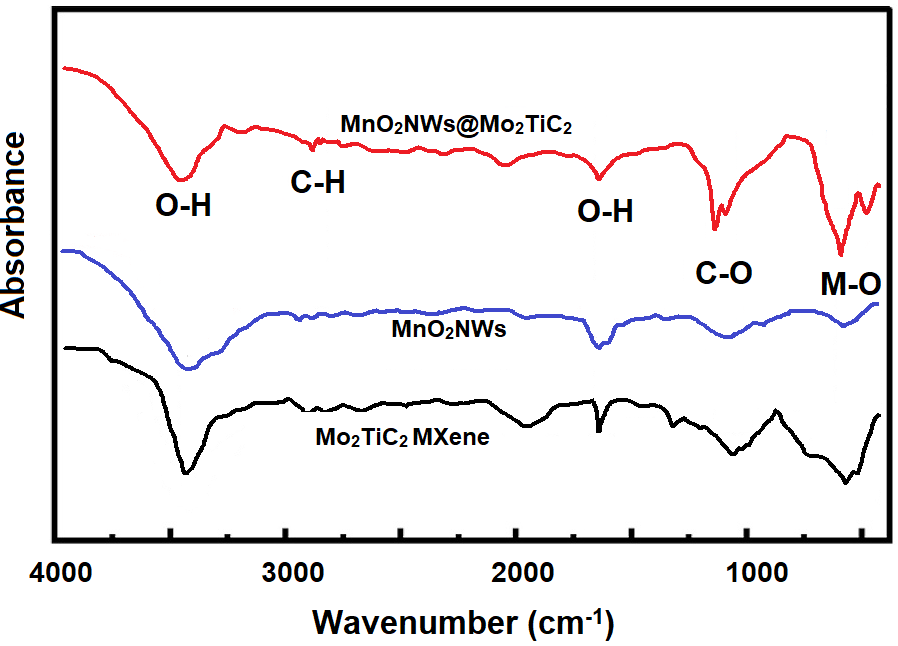
Fig. S2** FTIR spectra of MnO_2_NWs@Mo_2_TiC_2_ nanocomposite, Mo_2_TiC_2_ MXene and MnO_2_NWs

**
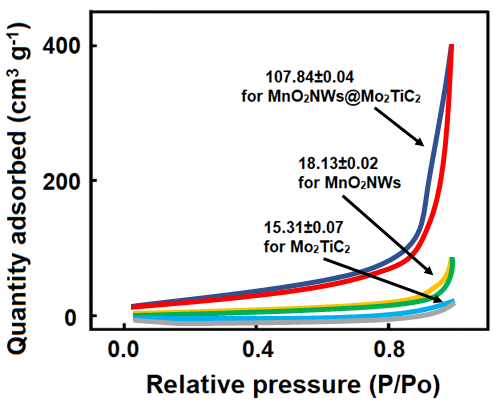
**

**Fig. S3** BET N_2_ adsorption-desorption isotherms of MnO_2_NWs@Mo_2_TiC_2_ nanocomposite, Mo_2_TiC_2_ MXene and MnO_2_NWs at 77K

**
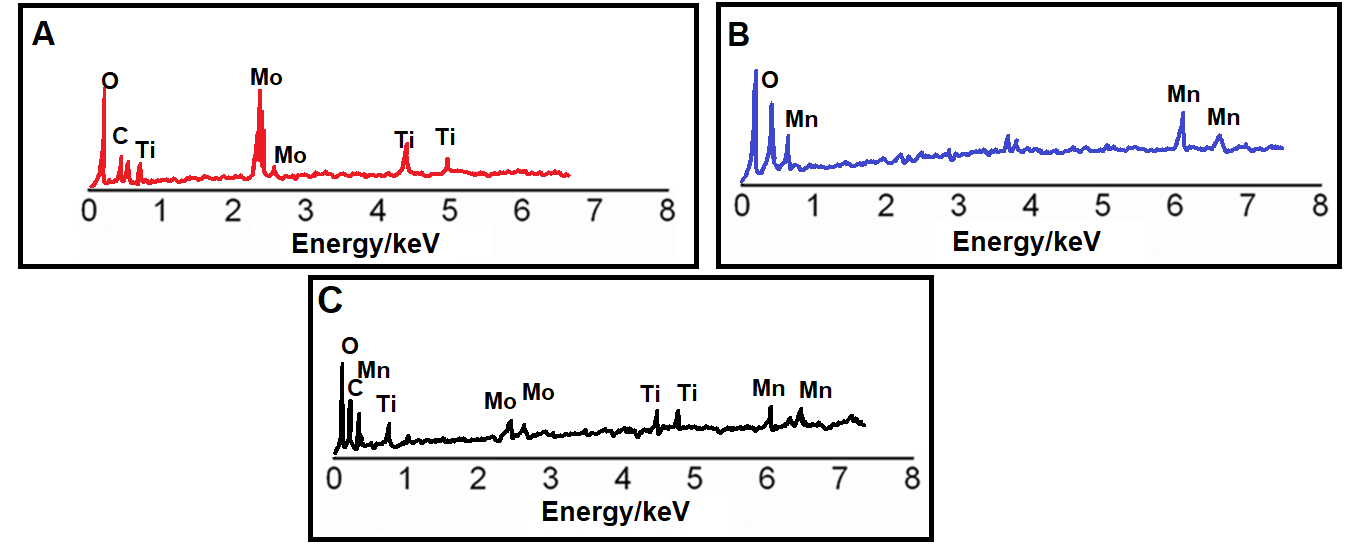
Fig. S4** EDX spectra of (A) Mo_2_TiC_2_ MXene, (B) MnO_2_NWs and (C) MnO_2_NWs@Mo_2_TiC_2_ nanocomposite

**
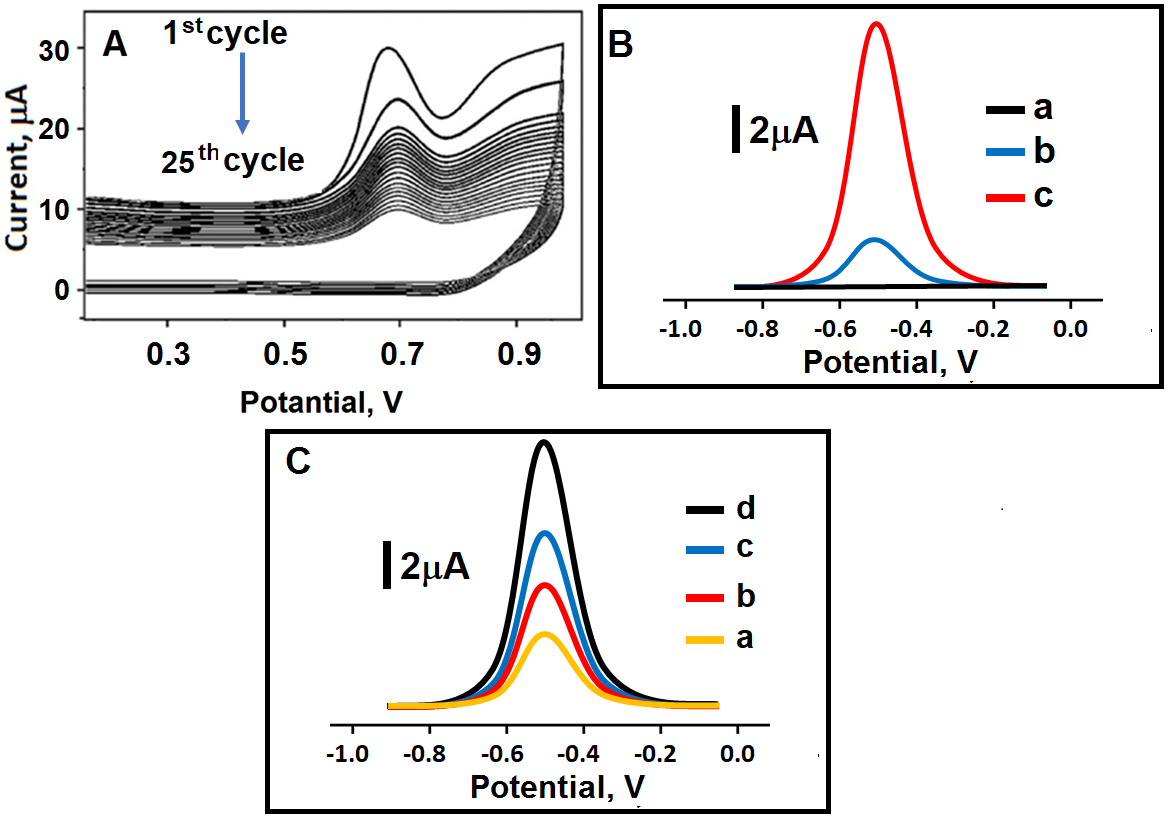
**

**Fig. S5** (A) 100.0 mmol L^-1^ Py polymerization containing 25.0 mmol L^-1^ FEN on MnO_2_NWs@Mo_2_TiC_2_ /GCE (Scan rate: 50 mV s^-1^); (B) SWVs of the prepared electrodes in this study: (a) MIP/MnO_2_NWs@Mo_2_TiC_2_/GCE in blank buffer solution (pH 4.5), (b) NIP/MnO_2_NWs@Mo_2_TiC_2_/GCE after rebinding of 10.0 nmol L^-1^ FEN in 0.1 M PBS (pH 4.5), (c) MIP/MnO_2_NWs@Mo_2_TiC_2_/GCE after rebinding of 10.0 nmol L^-1^ FEN in 0.1 M PBS (pH 4.5); (C) SWVs of different molecularly imprinting electrodes after rebinding of 10.0 nmol L^-1^ FEN in 0.1 mol L^-1^ PBS (a) MIP/bare GCE, (b) MIP/Mo_2_TiC_2_ MXene/GCE, (c) MIP/MnO_2_NWs/GCE, (d) MIP/MnO_2_NWs@Mo_2_TiC_2_ /GCE

**
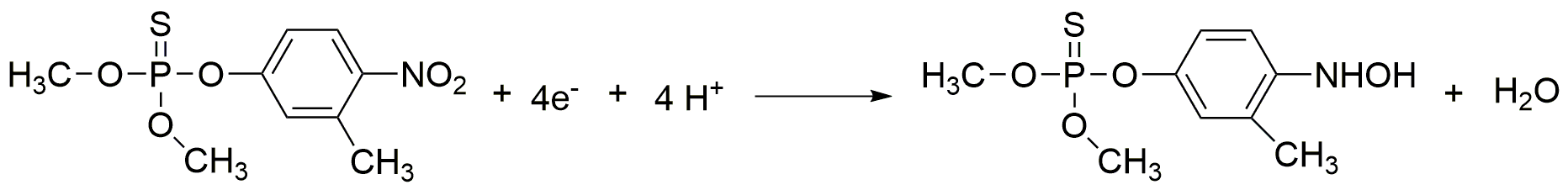
Fig. S6** The proposed electro-reduction for FEN on MIP/MnO_2_NWs@Mo_2_TiC_2_/GCE

**
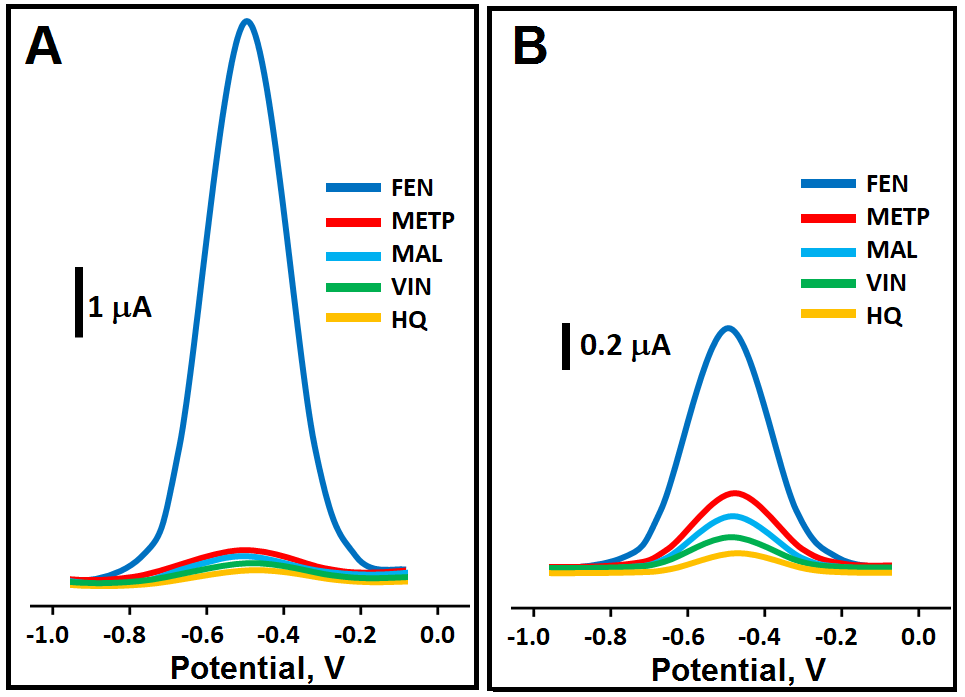
Fig. S7** SWVs of (A) MIP/MnO_2_NWs@Mo_2_TiC_2_/GCE and (B) NIP/MnO_2_NWs@Mo_2_TiC_2_/GCE in 10.0 nmol L^-1^ FEN, 1000.0 nmol L^-1^ METP, 1000.0 nmol L^-1^ MAL, 1000.0 nmol L^-1^ VIN and 1000.0 nmol L^-1^ HQ in 0.1 M PBS (pH 4.5)

**
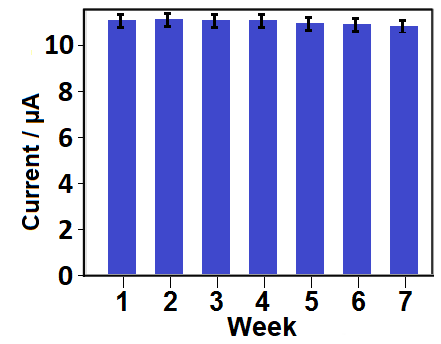
**

**Fig. S8** Stability test of MIP/MnO_2_NWs@Mo_2_TiC_2_/GCE including 10.0 nmol L^-1^ FEN by using SWV method (n=6)

**Table S1** Recovery results of FEN (n=6)

| **Sample** | **Added FEN**  **(nmol L^-1^)** | **Found FEN**  **(nmol L^-1^)** | **Recovery**  **(%)** |
| --- | --- | --- | --- |
| White flour | - | 0.44 ± 0.02 | - |
|  | 2.00 | 2.45 ± 0.04 | 100.41 ± 0.04 |
|  | 4.00 | 4.43 ± 0.05 | 99.77 ± 0.04 |
|  | 6.00 | 6.42 ± 0.01 | 99.69 ± 0.08 |

Recovery = Found FEN, nmol L^-1^ / Real FEN, nmol L^-1^

**Table S2** k and k′ values of FEN imprinted electrodes (MIP/MnO_2_NWs@Mo_2_TiC_2_/GCE and NIP/MnO_2_NWs@Mo_2_TiC_2_/GCE) (n=6)

|  | **MIP** | | **NIP** | |  |
| --- | --- | --- | --- | --- | --- |
|  | **∆i (µA)** | **k** | **∆i (µA)** | **k** | **k′** |
| FEN | 10.15 ± 0.02 | - | 1.00 ± 0.02 | - | - |
| METP | 0.50 ± 0.03 | 20.30 | 0.30 ± 0.01 | 3.33 | 6.10 |
| MAL | 0.40 ± 0.04 | 25.38 | 0.20 ± 0.06 | 5.00 | 5.08 |
| VIN | 0.30 ± 0.07 | 33.83 | 0.10 ± 0.05 | 10.00 | 3.38 |
| HQ | 0.20 ± 0.01 | 50.75 | 0.05 ± 0.02 | 20.00 | 2.54 |

Analyte concentrations: 10.0 nmol L^-1^ FEN, 1000.0 nmol L^-1^ METP, 1000.0 nmol L^-1^ MAL, 1000.0 nmol L^-1^ VIN and 1000.0 nmol L^-1^ HQ

k = ∆i _FEN_/∆i _interfering chemical_ and k′ = k_MIP/_k_NIP_
